# Pseudoexfoliative Syndrome in Cataract Surgery—A Quality Register Study and Health Economic Analysis in the Split-Dalmatia County, Croatia

**DOI:** 10.3390/jcm13010038

**Published:** 2023-12-20

**Authors:** Ivan Borjan, Robert Stanić, Ivna Pleština-Borjan, Maja Pavić, Silvia N. W. Hertzberg, Ljubo Znaor, Beáta Éva Petrovski, Goran Petrovski

**Affiliations:** 1 Clinical Department of Ophthalmology, University Hospital Center, 21000 Split, Croatia; ivan.borjan@kbsplit.hr (I.B.); robert.stanic@kbsplit.hr (R.S.); lznaor@kbsplit.hr (L.Z.); 2 University of Split, School of Medicine, 21000 Split, Croatia; ivna.plestina@gmail.com (I.P.-B.); pavicmajaa@gmail.com (M.P.); 3 Center for Eye Research and Innovative Diagnostics, Department of Ophthalmology, Institute for Clinical Medicine, Faculty of Medicine, University of Oslo, 0313 Oslo, Norway; s.n.w.hertzberg@medisin.uio.no (S.N.W.H.); b.e.petrovski@medisin.uio.no (B.É.P.); 4 Department of Ophthalmology, Oslo University Hospital, 0450 Oslo, Norway; 5 UKLONetwork, University St. Kliment Ohridski-Bitola, 7000 Bitola, North Macedonia

**Keywords:** pseudoexfoliation syndrome, cataract surgery, phacoemulsification, DRG, health economic analysis, Croatia

## Abstract

Purpose: To investigate the impact of pseudoexfoliation (PEX) syndrome on intraoperative phacoemulsification (PHACO) parameters and assess the economic cost of PHACO surgery for cataracts in patients with and without PEX syndrome. Methods: This was a retrospective quality register study on 5889 patients (6236 eyes) who underwent PHACO cataract surgery in the Eye Clinic, Clinical Hospital Centre Split, Croatia, over a 7-year period (May 2015 to December 2022), in accordance with the Guidelines of the Helsinki Declaration and approval from the Research Ethics Committee of the University Hospital Centre Split, Croatia. Inclusion criteria were patients with either presenile or senile cataract or cataract related to PEX syndrome who undertook PHACO procedure by the same experienced surgeon using the same PHACO device (Infiniti Vision System, Alcon Laboratories, Inc., Fort Worth, TX, USA). Eyes were categorized according to PEX presence- (PEX group) or absence (Group without PEX). The following recorded data about intraoperative PHACO parameters were collected: Cumulative Dissipated Energy (CDE), Ultrasound total time, PHACO time, torsional time, aspiration time, estimated fluid used, and duration of the surgical procedure. In the economic analysis, all PHACO parameters were considered, with a specific focus on the duration of the surgical procedure, costs associated with additional medical materials and devices, complications during surgery, and surgery procedure Diagnosis-Related Group (DRG) codes. Results: A total of 4535 cases were eligible for inclusion in the study, 278 (6.13%) were diagnosed with PEX and 4257 (93.87%) had no PEX. Significantly higher PHACO parameters were observed in the PEX group. Similarly, a statistically significant increase in the values of all PHACO parameters was observed with the increase in nuclear lens density. Intraoperative complications were more frequent in the PEX group. Zonular weakness requiring the use of a capsular tension ring (CTR) and posterior capsular rupture occurred 30 and 13 times more often, respectively, in the PEX group. The expected cost of the PHACO procedure was found to be 1.4 times higher in patients with PEX, compared to those without PEX, for all types of nuclear cataract. Conclusions: All PHACO parameters are significantly higher in patients with PEX. The costs associated with PHACO surgery for cataracts are greater for patients with PEX and are not covered by the present DRG codes, which highlights the need to accordingly adjust the DRGs for PHACO procedures in PEX patients, in order to maintain the quality of healthcare provided for these vulnerable patients.

## 1. Introduction

Pseudoexfoliation (PEX) syndrome is a systemic disorder primarily manifesting in ocular symptoms. It is characterized by accumulation of whitish fibrillary amyloid-like materials in various ocular structures, primarily in the anterior segment (corneal endothelium, iris-pupillary margin, anterior lens capsule, zonular fibers, and trabecular meshwork), as well as in connective tissue of different visceral organs [1,2,3,4].

The prevalence of PEX syndrome tends to increase with the age, but it is rare before age of 50 years. It is estimated that up to 20% of individuals over the age of 60 may be affected by this condition [1,3]. While PEX syndrome occurs worldwide, its prevalence significantly varies among different geographic regions and different ethnic groups. It is more common in Scandinavian, Mediterranean, and Middle Eastern countries (with a prevalence between 16% and 30%), and less common among the Inuit populations in Greenland, Alaska, and Canada (with a prevalence of 0%) [5,6,7,8,9].

The exact etiopathogenesis of this condition is not yet completely understood, but it is considered a multifactorial disorder that develops through a complex interplay of environmental factors, such as UV exposure and geographic latitude, dietary factors, viral infection and trauma involving the anterior segment of the eye, along with genetic predisposition. Current studies have shown that factors associated with oxidative stress and antioxidant defense mechanisms can play important roles in the etiopathogenesis of PEX [10,11]. Recently, mutations in the *lysyl oxidase-like 1* gene (*LOXL1*) at the locus 15q22 have been identified as the major contributing factor to the fibrotic disorder seen in PEX syndrome [12,13]. Many other gene variants or mutations can potentially be associated with the development of this condition, including the *clusterin* gene, *calcium voltage-gated channel subunit alpha 1* gene variants, *glutathione transferase* gene, *fibulin-5* gene, tumor necrosis factor-alpha, and tumor growth factor beta 1, as well as the presence of altered microRNA molecules, which regulate post-transcriptional gene expression, in the aqueous humor of individuals with PEX [3,14].

Eyes affected by PEX syndrome are at higher risk of developing glaucoma, and this condition has been correlated with an increased incidence of cataract formation [6,15]. Up to 44% of patients with PEX develop pseudoexfoliative glaucoma [16].

Phacoemulsification (PHACO) is a standard method of cataract surgery today, with significantly lower rates of intraoperative and postoperative complications compared to other older methods. Results continue to improve with the development of PHACO devices and surgical techniques. However, the presence of PEX can complicate this routine cataract surgery method.

Eyes affected by PEX syndrome are often characterized by weak response to mydriatic drops (due to fibrillary material deposits on the iris surface and the pupillary margin), as well as lens instability (due to fragmented and degenerated zonules) [6,17]. These alternations in the anterior eye segment tissue can make cataract surgery in eyes with PEX potentially challenging, necessitating the expertise of a skilled and experienced surgeon.

All these factors contribute to an increased rate of intraoperative complications, including zonular dehiscence (separation or weakening of the lens zonular fibers), posterior lens capsule rupture, vitreous loss, and nucleus drop [2,6,13,18,19,20]. Additionally, a higher rate of postoperative complications has been reported, including corneal edema and descemetitis, elevated intraocular pressure (IOP) spikes, inflammation, anterior capsule phimosis, posterior capsule opacification, and intraocular lens (IOL) dislocation, often within the capsular bag [6,13,21].

PEX syndrome is often associated with dense nuclear cataracts (hard cataracts), which require a higher amount of total ultrasound (US) energy for their removal [20,22,23]. This can lead to increased damage to intraocular structures, particularly corneal endothelial cells [23,24]. Additionally, the volume of fluidics and prolonged aspiration during the PHACO procedure can contribute to a higher rate of intraoperative complications, and may also have a significant impact on corneal endothelial cell loss [23].

Due to poor pupillary dilatation and zonular weakness, surgeons often need to employ additional procedures and devices during PHACO surgery, such as mechanical dilatation, visco-dilatation, pupil expansion devices, or a capsular tension ring (CTR) (a small ring-shaped medical device implanted into the capsular bag during cataract surgery to help maintain its shape and stability and prevent complications). All these procedures prolong the duration of cataract surgery in eyes with PEX, causing further tissue damage and potentially resulting in poor visual outcome. Additionally, they lead to a significant increase in cataract surgery expenses.

Modern PHACO machines offer the capability to record various critical parameters during cataract surgery, including Cumulative Dissipated Energy (CDE); US total time; PHACO, torsional, and aspiration time; as well as estimated fluid usage and duration of the surgical procedure.

The presence of narrow pupils, zonular dehiscence, and hard nuclei in eyes affected by PEX syndrome could potentially influence such PHACO device parameters. Very few studies have conducted an analysis of intraoperative parameters in patients with PEX syndrome undergoing PHACO surgery, which we investigate here [24,25,26]. There is a lack of information in the existing literature regarding any associated increase in cataract surgery costs in patients with PEX syndrome, so we also performed a health economic analysis for the real costs of PHACO surgery for cataracts in patients with PEX and compared them to costs for performing surgery in patients without PEX.

## 2. Methods

This was a retrospective quality register study analyzing data from 5889 patients (6236 eyes) who underwent PHACO cataract surgery by the same surgeon in the Eye Clinic, Clinical Hospital Centre Split—the largest healthcare provider in the Split-Dalmatia County in Croatia—over a 7-year period (May 2015 to December 2022).

Our research was conducted in accordance with the Guidelines of the Helsinki Declaration and received approval from the Research Ethics Committee of the University Hospital Centre Split, Croatia. To ensure transparent reporting, this study adhered to the Strengthening the Reporting of Observational Studies in Epidemiology (STROBE) guidelines.

For inclusion in the analysis, patients had either presenile or senile cataract or cataract related to PEX syndrome, and had undergone the PHACO procedure with implantation of various types of IOLs. Furthermore, the procedure had to be performed by the same experienced surgeon using the same PHACO device (Infiniti Vision System, Alcon Laboratories, Inc., Fort Worth, TX, USA) (Supplementary Figure S1).

We excluded patients with posttraumatic cataracts, patients with a history of any other previous eye surgery procedures, eyes with other ophthalmological diseases, patients with IOP higher than 23 mmHg, eyes that required a combined cataract and glaucoma surgery, cataracts in eyes with dislocated lenses, and cataracts in patients under 18 years of age. We excluded patients under the age of 18 to ensure consistency of the study because such patients belong to the pediatric population and PEX syndrome is extremely rare in this population group, while the condition is predominantly associated with older adults. Patients with incomplete data or those who underwent surgery with a different PHACO device other than the device used in the study were also excluded from the analysis. After all exclusion criteria were applied, 4535 cases were suitable for data analysis.

Data were collected and analyzed from two sources: patients’ medical records and surgical protocols stored at the study site. Demographic information, such as age and gender, as well as data regarding presence of PEX in the anterior eye segment, was retrieved from patients’ medical records and categorized as either “PEX present” (PEX group) or “PEX absent” (Group without PEX). The surgeon assessed the presence of PEX using a thorough eye examination with a slit lamp biomicroscope while the pupil was fully dilated. PEX presence was determined by the observation of fibrillary deposits on the pupillary margin, anterior lens capsule, or both.

Furthermore, data regarding the presence of phakodonesis or zonular instability were gathered from patients’ medical records and categorized as either “present” or “absent”. Information about the size of the pupil was extracted in a qualitative manner as “narrow” or “normal” pupils.

Lastly, data about the type of cataract and the grade of nuclear density were retrieved from patients’ medical records. The surgeon assessed the cataract nucleus density during preoperative slit-lamp examination with a dilated pupil, using the lens opacities classification system III (LOCS III) [27]: grade 0 for eyes without nuclear opacification, up to grade 6 for severe nuclear opacification and brown-red colored nucleus. Eyes were divided into two groups regarding nuclear density grade: lower nuclear cataract grade (grades 0 to 2) and higher nuclear cataract grade (grades 3 to 6).

The data about the course of the surgical procedure were collected from the surgical protocol. All surgical procedures were performed under topical anesthesia using the same phacoemulsification device. In brief, after paracentesis, visco-elastic was introduced into the anterior chamber, followed by a temporal corneal incision. A continuous curvilinear capsulorhexis was created using a cystotome, after which hydrodissection and hydrodelineation procedures were performed. The nucleus was extracted from the eye using the stop and chop technique. After employing automated irrigation-aspiration to remove the cortex, the capsular bag was filled with visco-elastic, and a foldable IOL was inserted into the capsular bag. Finally, the visco-elastic material was aspirated and incisions were hydrated; then, intracameral antibiotic was applied.

The following recorded data about the intraoperative PHACO parameters were also collected: CDE (percent seconds), US total time (seconds), PHACO time (seconds), torsional time (seconds), aspiration time (seconds), estimated fluid used (milliliters), and duration of the surgical procedure (minutes: seconds). Data regarding the duration of the surgical procedure refer to the time from the beginning of the use of the PHACO device to the end of the procedure. Therefore, the actual values for the surgical procedure duration are slightly longer than the listed values. Intraoperative PHACO parameters were analyzed and compared between the observed groups.

Furthermore, the data about intraoperative complications were retrieved from the surgical protocol and analyzed accordingly. Information about the use of capsular dye, CTR, and pupil expansion devices was also collected. Intraoperative complications were managed following established professional standards and documented accordingly. Iris retractors and mechanical or visco-dilatation techniques were used for narrow pupils. In instances of zonular instability, the CTR was placed in the capsular bag, and anterior vitrectomy was performed in cases of posterior capsule rupture with vitreous prolapse. When capsule rupture occurred, a three-piece IOL was most commonly placed in the ciliary sulcus, and in few cases, primary IOL implantation was deferred.

Descriptive statistical analysis was initially performed; the results are presented in percentages and median with interquartile range (IQR: Q3-Q1: 25th and 75th percentile) and range (difference between the highest and lowest values). The normality of continuous variables was tested on a histogram, a Q-Q plot, and using the Shapiro–Wilks or Kolmogorov–Smirnov tests. Due to the non-normal distribution of the continuous variables, the Mann–Whitney U test was used to detect median differences in continuous, numerical variables between the two groups (PEX and without PEX). The chi-square (χ^2^) test was used to test the differences in the distribution of categorical variables. All differences were considered significant at *p* < 0.05. Statistical Package for STATA (Stata version 17.0; College Station, TX, USA) was used for the statistical analyses.

Health economic cost analysis was performed to estimate the present Diagnosis-Related Group (DRG) costs and the expected DRG costs for the procedures that tend to take more time and resources. In addition, the expected loss due to the unaccounted costs in the DRG weights attached to these procedures was calculated. The analysis follows the cataract grading used in this study. We considered the base for our calculation to be the DRG cost of all types of cataracts, of EUR 601.64, from the Croatian Institute of Health Insurance (hzzo.hr/poslovni-subjekti/hzzo-za-partnere/sifranici-hzzo-0, accessed on 10 September 2023), and estimated the cost per minute of the procedure accordingly for the different type of cataracts in our study. This cost was then multiplied by the time taken to perform the PHACO procedure in all other cases. We then subtracted the product from the base DRG cost to obtain the estimated loss.

## 3. Results

### 3.1. Demographic Characteristics of the Study Population

Of 6236 eyes that underwent cataract surgery in the observed period, 4535 met the inclusion criteria of the study. PEX was diagnosed in 278 (6.13%) eyes (PEX group), and in 4257 (93.87%), no sign of PEX was present (Group without PEX).

Table 1 shows the demographic characteristics of all patients who underwent cataract surgery, as a whole and in relation to the presence of PEX. There was no significant association between PEX and gender (*p* = 0.099). The median age of all patients was 75 years (IQR: 68–80), and the age was significantly higher in the PEX group (median: 77; IQR: 72–81) than in the Group without PEX (median: 75 (IQR: 68–80)) (*p* < 0.001).

### 3.2. Intraoperative Parameters during PHACO Procedures in Patients with and without PEX

**Figure 1:**
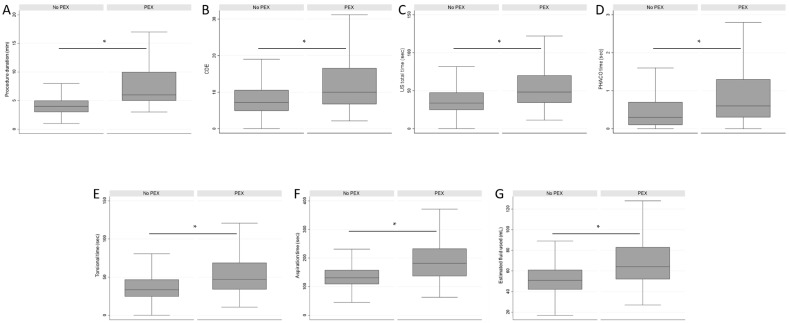
Comparison of the surgical parameters between the PEX group and the Group without PEX. (**A**): Procedure duration (min); (**B**): Cumulative Dissipated Energy (CDE); (**C**): Ultrasound (US) total time (sec); (**D**): PHACO time (sec); (**E**): Torsional time (sec); (**F**): Aspiration time (sec); (**G**): Estimated fluid used (mL). Data are presented with median, IQRs (upper and lower quartiles) and ranges (minimum and maximum); Mann–Whitney U test; Statistical significance shown is * *p* < 0.001.

In terms of procedure time/duration, the median for all PHACO procedures was 4 min (IQR: 4–5). However, in the PEX group, the procedure duration was significantly longer (median: 6; IQR: 5–10) compared to that of the Group without PEX (median: 4; IQR: 3–5) (*p* < 0.001) (Figure 1A).

Across all procedures, the median CDE was 7.3 (IQR: 4.9–10.9). In the PEX group, the median CDE was significantly higher (median: 9.98; IQR: 6.7–16.6) compared to that of the Group without PEX, (median: 7.2; IQR: 4.91–10.6) (*p* < 0.001) (Figure 1B).

The median US total time was 34.7 s (IQR: 25.2–48.9) and it was significantly higher in the PEX group (median 48.2; IQR: 34.3–70.2) than in the Group without PEX (median 33.8; IQR: 24.8–47.6) (*p* < 0.001) (Figure 1C).

In all procedures, the median PHACO time was 0.3 s (IQR: 0.1–0.7). The PEX group exhibited a significantly longer median PHACO time (median 0.6; IQR: 0.3–1.3) compared to the Group without PEX (median 0.3; IQR: 0.1–0.7) (*p* < 0.001) (Figure 1D).

The median torsional time was 34.2 s (IQR: 24.9–48.2) across all cases, but it noticeably increased in the PEX group (median: 47.2; IQR: 33.9–68.8) compared to the Group without PEX (median 33.4; IQR: 24.5–46.9) (*p* < 0.001) (Figure 1E).

Regarding aspiration time, the overall median was 133 s (IQR: 111–161). In the PEX group, it was significantly prolonged (median: 181.5; IQR: 137–233) compared to in the Group without PEX (median: 131; IQR: 109–158) (*p* < 0.001) (Figure 1F).

In terms of estimated fluid usage, the overall median was 51 mL (IQR: 43–61). The PEX group again demonstrated a significantly higher fluid use (median: 64; IQR: 52–83) compared to that in the Group without PEX (median: 51; IQR: 42–61) (*p* < 0.001) (Figure 1G).

Furthermore, there was a statistically significant association between PEX syndrome and intraoperative floppy iris syndrome (IFIS) (*p* = 0.035). Otherwise, there was no significant association between PEX and dying of the anterior capsule (*p* = 0.583).

CTR was used in 3.96% of cases in the PEX group, which was much more common than in the Group without PEX (0.12%). Posterior capsule rupture occurred in 5.40% of surgical procedures within the PEX group compared to 0.42% in the Group without PEX.

### 3.3. Intraoperative Parameters during PHACO Procedures in Patients with Different Grade of Nuclear Cataract and Presence or Absence of PEX

**Figure 2:**
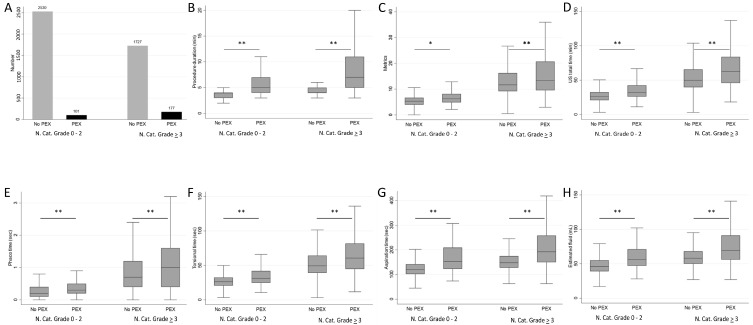
Comparison of the surgical parameters in relation to presence or absence of PEX and nuclear cataract grade. (**A**): Number of cases in relation to presence or absence of PEX and nuclear cataract grade; (**B**): Procedure duration (min); (**C**): Cumulative Dissipated Energy (CDE); (**D**): Ultrasound (US) total time (sec); (**E**): PHACO time (sec); (**F**): Torsional time (sec); (**G**): Aspiration time (sec); (**H**): Estimated fluid used (mL). Data are presented with median, IQRs (upper and lower quartiles) and ranges (minimum and maximum); χ^2^ test; Mann–Whitney U test; Statistical significance shown is * *p* < 0.01; ** *p* < 0.001.

An analysis was performed to examine the relationship between nuclear cataract grade and presence of PEX. Of the 4535 study participants, 2631 had a lower nuclear cataract grade (0–2). Among these, 2530 had no signs of PEX, while 101 were diagnosed with PEX. A higher nuclear cataract grade (≥3) was present in 1904 study participants, from which 1727 had no signs of PEX and 177 had PEX. The statistical analysis showed a significant association between the presence of PEX and nuclear cataract grade (*p* < 0.001) (Figure 2A).

For individuals with a lower nuclear cataract grade and without PEX, the median duration was 4 min (IQR: 3–4), while it was 5 min (IQR: 4–7) among those with lower nuclear cataract grade and PEX. Furthermore, for individuals with a higher nuclear cataract grade and without PEX, the median duration was 4 min (IQR: 4–5), while it was significantly higher, i.e., 7 min (IQR: 5–11), among those with higher nuclear cataract grade and PEX (*p* < 0.001) (Figure 2B).

Among individuals with a lower nuclear cataract grade and without PEX, the median CDE was 5.33 (IQR: 3.98–6.67), and for those with a lower nuclear cataract grade and PEX, it increased to 6.25 (IQR: 4.87–8.15). In contrast, individuals with a higher nuclear cataract grade and without PEX had a median CDE of 11.66 (IQR: 9.20–16.23), while those with both higher nuclear cataract grade and PEX had a significantly higher median CDE of 13.28 (IQR: 9.54–20.66) (*p* < 0.01 and *p* < 0.001) (Figure 2C).

The median US total time was 26.5 s (IQR: 20.9–32.7) among eyes with lower nuclear cataract grade and without PEX, which increased to 31.9 (IQR: 26.1–42.6) among those with lower nuclear cataract grade and PEX. Meanwhile, individuals with higher nuclear cataract grade and without PEX had a median US total time of 49.9 s (IQR: 39.7–65.5), while in those with both a higher nuclear cataract grade and PEX, the US time was significantly higher—62.5 s (IQR: 45.8–84.0) (*p* < 0.001) (Figure 2D).

The median PHACO time was 0.2 s (IQR: 0.1–0.4) among participants with lower nuclear cataract grade and without PEX, while it was 0.3 s (IQR: 0.2–0.5) among those with lower nuclear cataract grade and PEX. Among individuals with higher nuclear cataract grade and without PEX, the median PHACO time was 0.7 s (IQR: 0.4–1.2), and among those with higher nuclear cataract grade and PEX, it was significantly higher—1.0 s (IQR: 0.4–1.6) (*p* < 0.001) (Figure 2E).

The median torsional time also demonstrated variations across the groups. For individuals with a lower nuclear cataract grade and without PEX, the median time was 26.3 s (IQR: 20.7–32.4), and among those with a lower nuclear cataract grade and PEX, it increased to 31.3 (IQR: 24.8–42.0). In contrast, individuals with a higher nuclear cataract grade and without PEX had a median torsional time of 49.3 s (IQR: 39.1–64.2), while those with both a higher nuclear cataract grade and PEX had a significantly higher median torsional time of 60.6 s (IQR: 44.8–81.6) (*p* < 0.001) (Figure 2F).

For individuals with a lower nuclear cataract grade and without PEX, the median aspiration time was 120 s (IQR: 102–142), and for those with a lower nuclear cataract grade and PEX, it increased to 153 (IQR: 123–209). Meanwhile, individuals with a higher nuclear cataract grade and without PEX had a median aspiration time of 148 (IQR: 128–175), while those with both a higher nuclear cataract grade and PEX had a significantly higher median aspiration time of 192 s (IQR: 150–258) (*p* < 0.001) (Figure 2G).

Finally, the median estimated fluid usage for individuals with a lower nuclear cataract grade and without PEX had a median of 46 mL (IQR: 39–55), and among those with a lower nuclear cataract grade and PEX, it increased to 56 mL (IQR: 47–71). Meanwhile, individuals with a higher nuclear cataract grade and without PEX had a median fluid usage of 58 mL (IQR: 50–68), while those with both a higher nuclear cataract grade and PEX had a statistically higher median fluid usage of 69 mL (IQR: 56–91) (*p* < 0.001) (Figure 2H).

**Figure 3:**
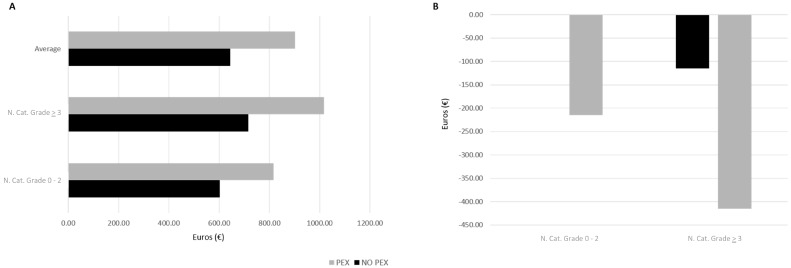
Expected DRG costs and losses for different nuclear cataract grades among patients with pseudoexfoliation (PEX) and without (NO PEX). (**A**): Expected DRG cost in euros (€) for PHACO procedure for different nuclear cataract grades among patients with pseudoexfoliation (PEX) and without (NO PEX); (**B**): Calculated loss in euros (€) for different nuclear cataract grades among patients with pseudoexfoliation (PEX) and without (NO PEX).

In the health economy cost analysis, the DRG cost for nuclear cataract grade 0–2 with no PEX was derived by dividing the general cataract cost of EUR 601.64 by the time of surgery for such a grade of cataract (4.2 min), equaling EUR 143.25 per minute. Consequently, nuclear cataract grade 0–2 with PEX resulted in an expected DRG cost of EUR 816.51, while grade ≥ 3 without and with PEX resulted in an expected DRG cost of EUR 716.24 and EUR 1017.06, respectively. On average, the expected DRG was found to be EUR 644.61 and EUR 902.46 for absence or presence of PEX, respectively (Figure 3A).

Considering the baseline DRG costs, the health institution appears to face losses performing cataract procedures on eyes with higher nuclear grade (≥3) despite PEX presence (ranging from a loss of EUR 115 to EUR 415 per patient), and also on eyes with PEX of lower grade of nuclear cataract amounting to EUR 215 loss per patient (Figure 3B).

## 4. Discussion

While PHACO is typically a routine surgical procedure, it can become more challenging in eyes with PEX, where there is a much higher risk of complications that may require additional interventions and use of specialized adjunctive devices.

This study focuses on assessing the impact of PEX syndrome on intraoperative PHACO parameters. Additionally, to the best of our knowledge, this is the first study to estimate the cost of PHACO procedures for cataracts in patients with PEX syndrome, and to compare it with the costs for the same procedure in patients without PEX.

Other studies have reported varying rates of PEX among patients with a cataract, ranging from 0.3% in Poland to 3.5% in France, 18% in Norway, and 33% in Finland, compared to ours (6.13%) [5,28,29,30]. It is important to note that the results can vary based on the overall prevalence of PEX for the specific population.

Our findings are consistent with previous research, which has demonstrated an age-related increase in the occurrence of cataracts among individuals with PEX syndrome [5,9]. The prevalence was only 0.4% in patients under 50 years of age and 82% in patients over 70 years.

Although our study indicated a slight predominance of PEX syndrome in males compared to females, this difference was not statistically significant. There are conflicting data, however, regarding sex predilection for PEX in the literature [5]. Some studies have reported a slight predominance of females among those with PEX syndrome (interpreting this as a reflection of longer lifespan in women), while others have reported the opposite [5,6,31,32,33].

The consequences of PEX syndrome on the anterior segment of the eye (nuclear dense cataract, zonular weakness, and narrow pupil) may also have an impact on the intraoperative PHACO parameters. In our study, all the parameters were significantly higher in the group of cases with PEX syndrome. The elevated values of CDE, US total time, PHACO time, and torsional time in the PEX group can be attributed to the presence of harder nuclear cataracts in this group, which consequently necessitate increased US energy for fragmentation. The amount of this energy exposure during PHACO is quantified using CDE, which represents the total energy dissipated in the anterior chamber during foot-pedal position 3 [34,35]. The degree of ultrasonic exposure during PHACO is an important determinant of corneal endothelial cell loss [23,24,34]. The values of aspiration time and the estimated amount of fluid used were 39% and 25% higher, respectively, in the PEX group compared to the Group without PEX. This, along with the CDE levels, can affect the postoperative state of the cornea [23]. Denser cataracts demand higher CDE and more time in foot-pedal position 3, resulting in a higher flow rate and fluid volume passing through the eye, which could increase the risk of corneal damage [36]. Moreover, heightened fluctuations, particularly when combined with a constricted pupil, can intensify the strain on the already weakened zonules, potentially leading to additional complications, such as zonulolysis and posterior capsule rupture.

In the PEX group, we observed over 30-times higher occurrence of zonular weakness (zonular instability) compared to the Group without PEX. Consequently, the use of a CTR was necessary in these eyes. Other studies have also reported a higher percentage of zonulopathy in the group of patients with PEX [19]. In four (1.44%) cases among our patients with PEX syndrome, the rupture of the posterior capsule and related complications prevented the primary implantation of an IOL. As a result, another secondary procedure for IOL lens implantation became necessary.

We also investigated the impact of nuclear density on all intraoperative PHACO parameters; it was found that as the grade of nuclear hardness increases in eyes with PEX, the values of all parameters significantly rise.

The total surgical procedure duration was found to be significantly prolonged in the PEX group compared to the Group without PEX. The median procedure duration in the PEX group was 2 min longer compared to the median in the Group without PEX. This means that during the time taken for one surgical procedure in a patient with PEX, a surgeon could perform approximately 1.5 cataract surgeries in eyes without PEX. We attribute this extended duration primarily to factors such as nuclear hardness, zonular weakness requiring CTR insertion, insufficient pupil dilation, and potential intraoperative complications, most notably posterior capsule rupture, with or without vitreous prolapse. The rupture of the posterior capsule of the lens was approximately 13 times more frequent in subjects with PEX than in those without it.

A limited number of studies have examined the differences in these parameters between patients with and without PEX syndrome [24,25,26,37]. Aygun et al. reported statistically significant longer US total time, PHACO time, and aspiration time, and a higher amount of estimated fluid usage in patients with PEX compared to those without PEX [25]. No differences could be observed in other parameters in the latter study, including CDE. These parameters suggest the comparable hardness of the nucleus in both study groups could be the underlying reason for this [25]. However, the overall procedure duration was not investigated in this study. In contrast, the study by Potemkin et al. demonstrated statistically significant higher CDE in patients with PEX in comparison to those without PEX syndrome [26]. The amount of aspirated BSS and duration of surgery were slightly higher in the PEX group, but not statistically significant. Kaljurand et al., consistently with our results, also found longer US total time and a higher amount of estimated fluid usage [24]. They explained this by the need for more cautious surgery in the PEX group due to zonular weakness and inadequate mydriasis.

All intraoperative PHACO parameters in our study were lower than those reported in other studies, including duration of the procedure [25,26]. Our surgical procedures were all performed by an experienced and skilled surgeon with over 20 years of experience, who has conducted thousands of PHACO surgeries. As a result, our procedures had naturally shorter durations/lower intraoperative PHACO parameters compared to those performed by less experienced surgeons. Additionally, the stop and chop technique was used for fragmenting hard nuclear cataracts to minimize CDE and potential cornea endothelial cell loss.

The results of our study indicate an increased risk of intraoperative complications during PHACO surgery for cataracts among patients with PEX syndrome, potentially resulting in poorer visual outcomes. The increased incidence of intraoperative complications, coupled with the prolonged duration of PHACO surgery, significantly inflate the overall costs of the procedure. Moreover, it may lead to additional expenses related to managing postoperative complications, including the possibility of secondary surgical interventions.

This study is the first to estimate the cost of performing PHACO surgery procedures for cataracts in patients with PEX syndrome compared to the cost of the same procedure in patients without PEX. We found that the overall costs per patient for cataract PHACO surgery procedures were higher in patients with PEX compared to those without PEX.

In Croatia, the cost of all PHACO procedures is assumed the same regardless of the severity of the cataract, the duration of the procedure, potential complications, or additional equipment and devices used (e.g., CTR, which accounts for EUR 103.39 or about one-sixth of the total procedure cost). The extended duration of surgeries in PEX patients and the presence of higher-grade nuclear cataracts with or without PEX prolongs the waiting list time for surgery. Surgeons in smaller centers currently tend to avoid operating on eyes with PEX and/or higher nuclear grade cataracts due to the higher risk of complications and poorer visual outcomes. Instead, they refer these patients to larger centers with more experienced surgeons, incurring additional costs related to travel, or even further delaying the surgical procedure. This ultimately leads to cataract worsening and an increase in complication rates, which consequently result in higher costs incurred.

Since there are no specific DRG codes to compensate for additional costs in PHACO surgery for cataracts in patients with PEX syndrome and/or higher-grade nuclear cataracts, this can lead to unintended DRG consequences, such as cost-cutting or losses, and patients not receiving the necessary additional medical resources for cataract management. Adjusting the weight of DRGs for PHACO surgeries for cataracts in eyes with PEX syndrome and/or higher-grade nuclear cataracts could result in an improved quality of care.

DRG systems are based on various national calculations and policies that reflect treatment costs. As new technologies emerge and new treatments are discovered, the DRG weights associated with previous treatments can become outdated. Therefore, generating new or updated DRG codes should be considered [38].

All of the above factors should be taken into consideration when determining the cost of PHACO surgery for different grades of cataracts in eyes with or without PEX syndrome. The expected cost of the PHACO procedure was found to be 1.4 times higher in patients with PEX compared to those without PEX for all types of nuclear cataract.

A limitation of our study is its retrospective nature, which limits the ability to establish causality between variables. In addition, we could not comprehensively monitor postoperative complications to calculate the total increase in costs for patients with PEX. Furthermore, we lacked data about pupil diameter in millimeters, and thus could not show its exact impact on the PHACO parameters.

## 5. Conclusions

All PHACO parameters proved to be significantly higher in patients with PEX syndrome, and PHACO surgery for cataracts is far more complex in these patients, thus requiring an experienced or skillful surgeon. The procedure duration is notably longer and complications are much more common in PEX patients; therefore, the costs associated with their surgical procedure are substantially greater and largely not covered by the present DRG reimbursement codes. It is important to emphasize that, currently in Croatia, there is the same DRG for all eyes with a cataract, regardless of whether or not PEX syndrome is present and regardless of the grade of nuclear cataract. Our results highlight the need to adjust the DRGs for PHACO procedures in PEX patients and different grades of nuclear cataract accordingly, in order to maintain the sustainability and quality of the healthcare provided for this vulnerable group.

## Figures and Tables

**Table 1:** Demographic characteristics of the patients overall and in relation to presence of PEX.

		Total *n* = 4535	PEX *n* = 278	Without PEX *n* = 4257	*p*
			*n* (%)		
Sex	Male	2148 (47.4)	145 (52.2)	2003 (47.4)	0.099
	Female	2387 (52.6)	133 (47.8)	2254 (52.6)	
Age (years)	Median (IQR)	75 (68–80)	77 (72–81)	75 (68–80)	<0.001
	Range	21–97	47–97	21–97	

χ^2^ test; Mann–Whitney U test, *p* < 0.05 being considered significant, PEX = Pseudoexfoliation syndrome, IQR = Interquartile range.

## Data Availability

The authors possess all primary data and agree to allow the journal to review the data upon request.

## Supplementary Materials

The following supporting information can be downloaded at: https://www.mdpi.com/article/10.3390/jcm13010038/s1. Figure S1. Participant flow diagram with inclusion/exclusion criteria.
