# Supplementary figures and images for: Pseudoexfoliative Syndrome in Cataract Surgery—A Quality Register Study and Health Economic Analysis in the Split-Dalmatia County, Croatia
Source: J Clin Med. 2023 Dec 20;13(1):38. doi: 10.3390/jcm13010038 (PMC10780027; doi:10.3390/jcm13010038)

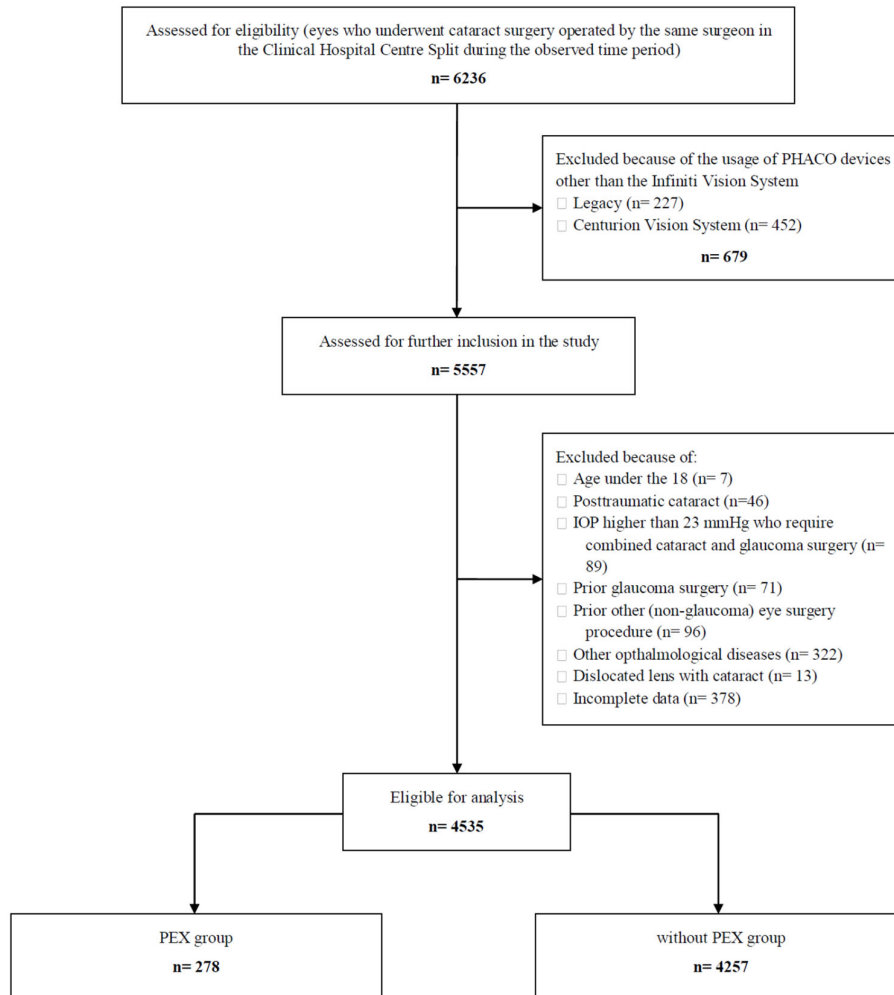

**Figure S1.** Participant flow diagram with inclusion/exclusion criteria.

Supplement: Supplementary file 1 [file jcm-13-00038-s001.zip › jcm-2663548-supplementary.pdf]
